# Utilizing Chicken Egg White and L-Cysteine for Green Synthesis of Carbon Dots: Rapid and Cost-Effective Detection of Cu^2+^ Ions

**DOI:** 10.3390/ma18030637

**Published:** 2025-01-31

**Authors:** Pablo Eduardo Cardoso-Ávila, Juan Luis Pichardo-Molina

**Affiliations:** Centro de Investigaciones en Óptica, Loma del Bosque 115, Leon 37150, Guanajuato, Mexico; jpichardo@cio.mx

**Keywords:** albumin, chicken egg white, l-cysteine, carbon dots, green chemistry, organic nanoparticles

## Abstract

A novel green synthesis method was developed for sulfur-doped carbon dots (S-C-dots) using chicken egg white (CEW) and L-cysteine for the rapid and cost-effective detection of copper ions (Cu^2+^) in water. This one-pot, room-temperature, base-catalyzed approach eliminated the need for energy-intensive processes and purification steps, adhering to the principles of green chemistry. The synthesized S-C-dots were characterized using UV–Vis, FT-IR, fluorescence, EDS, XRD, and Z-potential analyses. Among the six samples tested, A060 demonstrated superior properties, achieving a detection limit of 3.3 ppb (52 nM) for Cu^2+^ in aqueous solutions. This study highlights the potential of S-C-dots as eco-friendly, accessible, and efficient tools for monitoring heavy metal contamination in drinking water, offering a promising solution to global water safety challenges.

## 1. Introduction

Among the various natural and anthropogenic contaminants impacting soils, aquatic environments, and food sources, heavy metal pollution is a global concern with severe consequences for human health and the environment. Heavy metals have been detected not only in soils and water bodies but also in food sources, including crops, meat, and dairy products [1,2,3]. The lack of clean, potable water affects billions of people worldwide and represents a critical issue that must be addressed, as consumption of contaminated water leads to numerous diseases and deaths each year [4,5]. Heavy metal ions can cause significant health problems, including liver and kidney damage, skin disorders, cognitive impairment, and even cancer [4].

Copper is an essential industrial material valued for its excellent mechanical, electrical, and thermal properties. In the human body, copper is one of the necessary trace elements and ranks as the third most abundant metal. Copper deficiency can hinder enzyme activity and impair cellular metabolism, whereas an excess can lead to copper accumulation in the liver, potentially resulting in ascites, cirrhosis, and other liver-related conditions [6,7].

Given that water quality issues are often exacerbated in low-income regions, it is crucial to develop simple, rapid, and cost-effective methods for Cu^2+^ detection, as current techniques typically involve complex sample preparation and require costly, sophisticated instrumentation. Fluorescence spectrophotometry has emerged as a preferred approach due to its high sensitivity and selectivity, ease of use, and low cost compared to alternative methods.

Small fluorescent carbon nanoparticles (<10 nm) known as carbon dots (C-dots) have attracted considerable attention due to their chemical, physical, and optical properties. C-dots have been effectively used in the detection of various metal ions, including Al^3+^, Ag^+^, Au^3+^, Cd^2+^, Cr^3+^, Cu^2+^, Fe^3+^, Hg^2+^, Pb^2+^, and Pt^4+^, among others [8]. Most reported methodologies for the synthesis of carbon dots (C-dots) rely on the hydrothermal approach due to its simplicity [6,9,10,11,12]. However, this method is associated with high energy consumption, as it requires elevated temperatures and prolonged reaction times. Alternative methods, such as carbonization and pyrolysis, similarly demand extended thermal energy usage [13,14,15,16]. The carbon sources employed for C-dot synthesis are diverse, encompassing organic chemical reagents, waste biomass, and combinations of both, with each presenting specific advantages and limitations.

It is a common misconception that utilizing biomass as the sole carbon source inherently renders a synthesis method “green”. In some cases, the pretreatment of biomass and subsequent purification of the nanomaterial increase solvent consumption, reduce atom economy, and generate additional waste. Consequently, such methods may not fully adhere to the principles of green chemistry [17].

In contrast, for a nanomaterial designed as a metal sensor, alignment with the principles of green analytical chemistry is highly desirable [18]. This alignment can be achieved by implementing strategies such as employing direct analytical techniques, minimizing sample preparation, and reducing waste and energy consumption. A “greener” nanomaterial-based metal sensor can thus be developed by carefully selecting biomass sources that facilitate synthetic routes with minimal external energy requirements and by avoiding purification steps and specialized application conditions.

In recent years, our research group has focused on synthesizing C-dots from the readily available bovine serum albumin (BSA) protein. Initially, we produced blue-emitting C-dots with a fluorescence quantum yield (QY) of 16% using a two-step solvothermal method [19]. We later reported one-pot, room-temperature synthesis of C-dots using BSA in a water/ethanol solution, yielding yellow-emitting C-dots with an improved QY of 27% [20]. Our method further evolved with nitrogen doping and the elimination of ethanol in the synthesis, which increased the QY to 31% [21]. With each improvement, the C-dots exhibited better luminescent properties while reducing energy and reactant use, thus becoming “greener.” Additionally, we explored alternative biomass sources. Chicken eggs, which are abundant and cost-effective and have a lower carbon footprint than cattle, were used as a protein source. Employing food-grade chicken egg white (CEW) powder in C-dot synthesis resulted in a QY of 26% while reducing the time and cost and improving mass and solvent economy, making the process notably “greener” [22].

In this study, we present a novel synthesis method for sulfur-doped carbon dots (S-C-dots) for the direct, rapid, and selective detection of Cu^2+^ ions. CEW and L-cysteine were used as carbon sources in a one-pot, room-temperature, base-catalyzed synthesis method that was rapid, simple, and required no specialized equipment. S-C-dots were characterized using UV–Vis, FT-IR, fluorescence spectroscopies, EDS, XRD, and Z-potential analyses. Additionally, direct Cu^2+^ detection was achieved in an aqueous medium without any S-C-dot purification, yielding a limit of detection (LOD) of 3.3 ppb (52 nM). These features make this nanomaterial a promising candidate for direct, rapid, and accessible assessments of drinking water safety. The synthesis and analytical methods align with the principles of green chemistry and green analytical chemistry [17,18] due to their low energy use, simplicity, and direct application.

## 2. Materials and Methods

Six C-dot samples were prepared with L-cysteine-to-CEW ratios (*w*/*w*) ranging from 0% to 100%. Briefly, 400 mg of food-grade CEW powder (MCS, Puebla, Mexico) was added to 80 mL of Milli-Q water under magnetic stirring until the solution became clear. L-cysteine (0, 80, 160, 240, 320, or 400 mg) and 3 mL of 1 M NaOH were then mixed into the CEW/L-cysteine solution and allowed to react for 5 min. Finally, 1.6 mL of 25% glutaraldehyde was added, and the solutions were placed on a rotary shaker for 6 h. No further purification was required. The samples were labeled A000, A020, A040, A060, A080, and A100 according to the L-cysteine-to-CEW ratio.

UV–Vis and FT-IR spectra were acquired using an EPP2000 fiber-optic spectrometer (Stellarnet, Tampa, FL, USA) and a Cary 660 spectrometer (Agilent Technologies, Santa Clara, CA, USA). Z-potential measurements were conducted on a Zetasizer Nano-ZS (Malvern Panalytical, Great Malvern, UK). Energy-dispersive X-ray spectroscopy (EDS) was performed using a JEOL JSM-7800F (Tokio, Japan) scanning electron microscope. X-ray diffraction patterns were obtained using XRD D2 Phaser Bruker equipment (Billerica, MA, USA) with a Bragg–Brentano geometry and Cu-kα radiation (λ = 1.5418 Å). Fluorescence maps were obtained with an FS5 fluorometer (Edinburgh Instruments, Livingston, UK), using 2 and 4 nm bandwidths for excitation and emission, respectively. Fluorescence quantum yield (QY) values were measured using an SC30 integrating sphere module; spectra were recorded within the 520–700 nm range with a 530 ± 6 nm excitation bandwidth and a 0.25 nm emission bandwidth. For the Cu^2+^ dose–response curve, sample A060 was diluted 1:20 in Milli-Q water, and 0.5 mL of this diluted S-C-dot solution was mixed with 1.5 mL of a Cu^2+^ solution at concentrations ranging from 0.1 to 20 ppm. Each sample was prepared in triplicate, and fluorescence was measured from 545 to 600 nm with 530 nm excitation.

Consistent with the practices in the field, we utilized a single batch of food-grade CEW powder for this study. This approach is widely accepted in biomass-based research where the primary goal is to establish proof-of-concept methodologies. Testing multiple batches or brands, while potentially valuable, was beyond the scope of this study.

## 3. Results and Discussion

### 3.1. S-C-Dot Characterization

Following synthesis, the pH of each S-C-dot sample was tested using reactive strips, and all samples exhibited a pH of 7. The S-C-dot samples with L-cysteine fractions from 0% to 60% displayed high zeta-potential values (−42.2 ± 2.7, −42.8 ± 2.3, −41.2 ± 0.4, and −41.2 ± 3.9 mV, respectively), suggesting good colloidal stability. However, samples A080 and A100 had lower surface charges (−23.6 ± 3.6 and −16.6 ± 1.6 mV), which could have compromised their long-term stability, particularly A100, whose zeta potential suggested imminent agglomeration.

**Figure 1:**
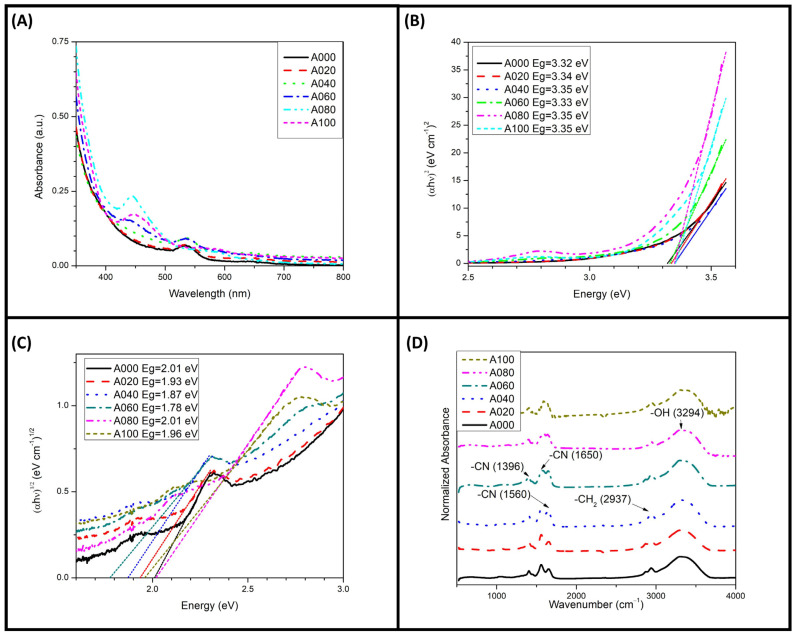
The (**A**) UV–Vis absorption, (**B**) direct and (**C**) indirect Tauc plots, and (**D**) FT-IR spectra of the S-C-dots. Doted lines in (**B**,**C**) indicated the fits to the linear segment of the curves.

All S-C-dot samples were intensely colored with no visible scattering. UV–Vis absorbance spectroscopy performed on diluted samples (1:10) revealed a broad absorption band below 400 nm in all samples, which was attributed to the π–π* transitions present in the S-C-dots [9,13,16,21] (Figure 1A). Additionally, samples A000 to A060 exhibited an absorption band around 530 nm, consistent with previously reported CEW-only C-dots [22]. In contrast, samples A080 and A100 displayed a new absorption band at 450 nm, accompanied by a marked decrease in the 530 nm band. The absorption bands above 400 nm originated from n–π* surface-state transitions associated with functional groups such as C=O, C=N, or heavily graphitic nitrogen [23,24,25].

Figure 1B,C show the energy band gaps of the S-C-dots, which were calculated using direct and indirect Tauc plots. The band gaps were determined as the x-axis intercepts of linear fits to selected segments of the curves. Notably, the addition of L-cysteine did not significantly alter the direct band gap of the S-C-dots, which remained between 3.32 and 3.35 eV (Figure 1B). These values were consistent with previously reported band gaps for C-dots [26].

However, the indirect band gaps varied with the L-cysteine concentration (Figure 1C). As L-cysteine was added during synthesis, the indirect band gap decreased from 2.02 eV in sample A000 to 1.78 eV in A060. The band gap increased to 2.02 eV in sample A080, then decreased again to 1.95 eV in A100. This behavior suggests that the addition of L-cysteine facilitated the formation of sulfur-rich functional groups on the S-C-dot surfaces, which initially reduced the indirect band gaps. At higher concentrations, colloidal stability was compromised, as evidenced by the zeta potential of A080, leading to S-C-dot agglomeration. This agglomeration increased the band gap and reduced the fluorescence intensities in these samples, as will be discussed in subsequent sections [27].

FT-IR analysis revealed identical vibrational modes across all samples (Figure 1D), indicating the presence of –OH, –CH_2_, –CO (amide I), –CN (amide II), and –CN (amide III) stretching modes [28,29].

**Figure 2:**
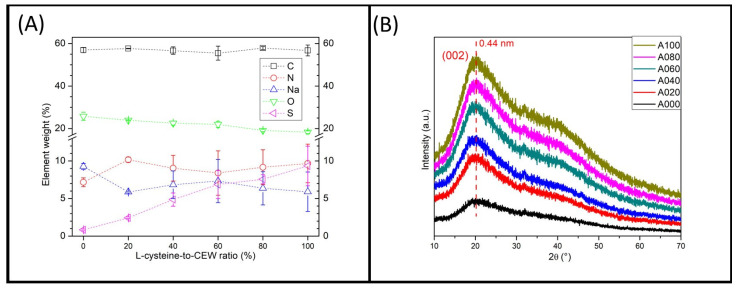
(**A**) The weight percentages of carbon, nitrogen, sodium, oxygen, and sulfur in the C-dot samples, as measured by EDS. (**B**) The XRD spectra of S-C-dots A000 to A100 (bottom to top).

Energy-dispersive X-ray spectroscopy (EDS) analysis revealed an increase in the sulfur content from 0.8% to 9.3% and a slight increase in the nitrogen content from 7.1% to 9.6% as the concentration of L-cysteine was raised (Figure 2A). Conversely, the oxygen content decreased from 25.8% to 18.3%. The relative contents of carbon and nitrogen remained stable at approximately 69% and 9%, respectively, across the samples. CEW is reported to contain minerals such as sulfur, potassium, sodium, and chlorine [30,31]. Therefore, the detection of sulfur in sample A000 was anticipated, and its level was expected to increase as the S-C-dots were supplemented with the thiol-containing amino acid L-cysteine.

The X-ray diffraction (XRD) data indicated low crystallinity in all S-C-dot samples; all patterns displayed a broad peak centered at 20.1°, corresponding to the (002) plane characteristic of graphitic materials (Figure 2B) [9,13,32,33]. The increased interlayer spacing (d = 0.44 nm) was attributed to low crystallinity, likely resulting from the incorporation of nitrogen-, oxygen-, and sulfur-containing groups [14,34].

**Figure 3:**
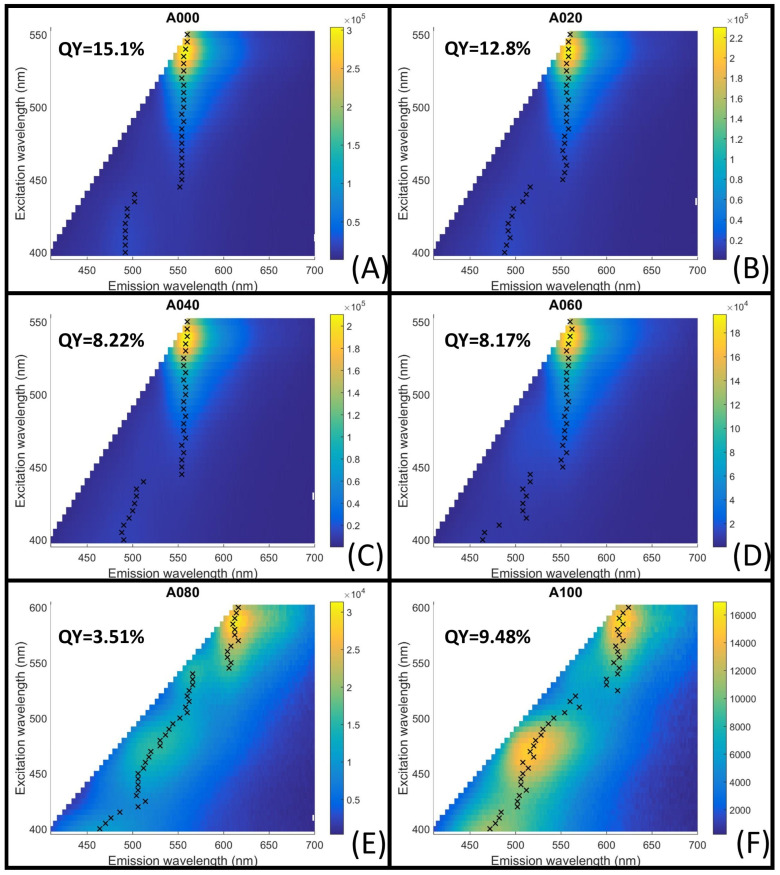
(**A**–**F**) Fluorescence maps of the S-C-dot samples, with black Xs indicating the maximum emission at each excitation wavelength.

Fluorescence analysis showed similar characteristics for samples A000 to A060, with low-intensity emissions at excitation wavelengths below 450 nm. A maximum emission wavelength centered at 555 nm was observed for excitations of 450 nm and above, with an excitation peak centered at 535 nm (Figure 3A–D). In contrast, samples A080 and A100 displayed a strong excitation-dependent emission maximum across the entire tested excitation range, along with considerably lower fluorescence intensities compared to the other S-C-dot samples. Since a clear emission maximum was not identified for these two samples, the excitation range was extended to 600 nm, where a maximum emission was observed for both at 610 nm with 590 nm excitation (see Figure 3E,F).

The fluorescence quantum yield (QY) for each C-dot sample is indicated in Figure 3. With the increasing L-cysteine content, the QY decreased from 15.1% in A000 to 8.17% in A060. Despite this reduction, A060 was selected as the optimal sample for fluorescence-based metal sensing, as it showed stable colloidal behavior, retention of the 530 nm absorption band, and consistent emission centered at 555 nm across a wide excitation range. Above all, it displayed a high sulfur content (8.2%), suggesting a greater abundance of thiol groups on the surfaces of the S-C-dots. Thiol groups are well known for their strong affinity toward metal ions. They form stable metal–thiol complexes that enhance the sensitivity and selectivity of metal detection. As such, A060 was identified as the most promising candidate for establishing the proof of concept explored in this study.

### 3.2. Performance of A060 as a Metal Sensor

**Figure 4:**
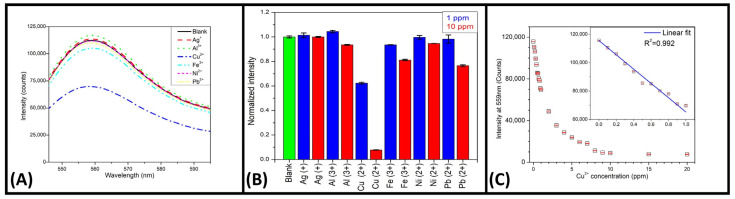
(**A**) The fluorescence spectra of A060 when combined with metals of interest at a concentration of 1 ppm. (**B**) The normalized fluorescence intensity of A060 at 559 nm in the presence of various metals at 1 and 10 ppm. (**C**) The fluorescence responses of A060 to Cu^2+^ at different concentrations, with the fluorescence at 559 nm shown as the means and standard deviations of three individual samples. Rectangles show the means, and red lines are the error bars. The inset displays the linear-response region of the curve (y = 115611 − 50404x).

A060 was evaluated as a metal sensor for Ag^+^, Al^3+^, Cu^2+^, Fe^3+^, Ni^2+^, and Pb^2+^. As shown in Figure 4A, these S-C-dots were sensitive to Al^3+^, Cu^2+^, Fe^3+^, and Pb^2+^ at 10 ppm, as indicated by a decrease in fluorescence intensity. However, only Cu^2+^ and Fe^3+^ were detectable at 1 ppm, with Cu^2+^ producing the strongest response. Figure 4B displays the dose–response curve for A060 when the Cu^2+^ concentration was increased up to 20 ppm; the fluorescence intensity at 559 nm decreased nearly exponentially. A linear model was fitted to the data in the 0 to 1 ppm range to calculate the limit of detection (LOD) and the limit of quantification (LOQ): LOD = 3δ/S = 3.3 ppb (52 nM) and LOQ = 10δ/S = 10.9 ppb (172 nM), where δ represents the standard deviation of the blank and S represents the slope of the linear model. The World Health Organization (WHO) sets a maximum limit of 2 ppm (31 µM) for Cu^2+^ in safe drinking water [35]; thus, the proposed fluorescence sensor can be used as a simple, rapid, and cost-effective tool for Cu^2+^ detection to ensure drinking water safety.

Table 1 summarizes previously reported C-dots used for Cu^2+^ detection, ordered from top to bottom with decreasing LOD, which range from 1.7 µM to 1 nM. The carbon sources have included chemical reactants (such as polyethyleneimine (PEI), citric acid, and amino acids) and various types of biomass, with no apparent correlation between the carbon source type and the achieved LOD. Most synthesis methods have involved high temperatures, external energy inputs, at least two purification processes, and specific buffer media for Cu^2+^ detection—all of which increase energy use and waste during the synthesis of the nanomaterials and their application. In contrast, our S-C-dots were synthesized using inexpensive CEW biomass and the readily available amino acid L-cysteine in a room-temperature method that required no external energy input and was completed in 6 h.

**Figure 5:**
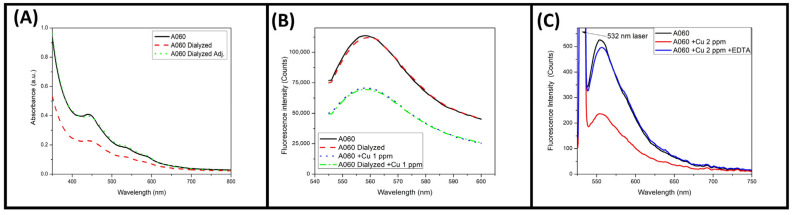
(**A**) The UV–Vis absorbance spectra of sample A060 as prepared and when dialyzed. (**B**) The fluorescence spectra of the as-prepared A060 and dialyzed A060 in the presence of 1 ppm Cu^2+^. (**C**) The fluorescence spectra of A060 diluted in water, diluted in 2 ppm Cu^2+^, and after the addition of 3 mg of EDTA.

Our S-C-dots are purification-free probes that are ready for Cu^2+^ detection without requiring a specific buffer medium. However, 20 mL of the A060 sample was dialyzed in 1 L of Milli-Q water for 48 h using a 1000 Da dialysis membrane. The washing liquid did not exhibit any coloration or fluorescence after the dialysis process was completed. Due to the dialysis process, the UV–Vis spectra of the dialyzed A060 showed slight dilution compared to the as-prepared A060 sample; this dilution could be adjusted to achieve the same absorbance in the fluorescence experiments, as shown in Figure 5A. The UV–Vis spectrum of the dialyzed sample did not show any significant changes, with the peak positions and widths remaining practically the same.

On the other hand, Figure 5B depicts the fluorescence spectra of both cases. The fluorescence spectra were virtually identical for the as-prepared and dialyzed A060 samples, as was the decrease in fluorescence when they were exposed to 1 ppm of Cu^2+^. These results clearly demonstrate that purification by dialysis is unnecessary, as it does not improve the sensing capabilities of the S-C-dots.

Figure 5C shows the fluorescence spectra of the A060 blank under 532 nm excitation and the quenching effect caused by 2 ppm of Cu^2+^. When 3 mg of the strong chelator ethylenediaminetetraacetic acid (EDTA) was added to the A060 + Cu^2+^ (2 ppm) sample, 96% of the fluorescence intensity was restored. This result suggests that chelation of Cu^2+^ by the functional surface groups on the S-C-dots brought the particles into close proximity, resulting in significant fluorescence quenching [43,44].

## 4. Conclusions

Fluorescent S-C-dots were synthesized by combining food-grade CEW powder with the readily available amino acid L-cysteine in varying mass ratios using a simple and rapid base-catalysis method at room temperature. The S-C-dots were characterized by UV–Vis, FT-IR, fluorescence, and EDS spectroscopy, which helped identify the most suitable sample for application as a metal sensor. A060, which was tested as a metal sensor without further purification, showed a decrease in fluorescence intensity when exposed to Fe^3+^ and Cu^2+^ at 1 ppm. However, as the response to Cu^2+^ was notably higher, a dose–response curve was generated specifically for this metal. The calculated limit of detection (LOD) and limit of quantification (LOQ) for Cu^2+^ were 3.3 ppb (52 nM) and 10.9 ppb (172 nM), respectively, indicating that A060 can serve as a simple, rapid, and cost-effective sensor for detecting Cu^2+^ to help ensure drinking water safety.

## Figures and Tables

**Table 1:** Comparison of previously reported C-dot-based fluorescent probes for detection of Cu^2+^.

Probe	Carbon Source (s)	Synthesis Method	Purification	Detection Medium	λex/λem	LOD (LR) [nM]	Ref.
DTPA-C-dots	TTDDA and citric acid	HT treatment at 180 °C for 6 h, DTPA Fun.	3500 Da DM for 72 h	pH 7.4 HEPES	340/446	NR (0–2600)	[9]
C-dots	Peanut shells	Pyrolysis at 400 °C for 4 h	FM, 1000 Da DM	Water	312/413	4800 (0–5000)	[33]
CPDs	Urine	Dehydration and carbonization at 200 °C for 12 h	C, FM, 1000 Da DM for 12 h	6.4 μM EDTA	450/510	1700 (0–30,000)	[13]
C-dots	Banana juice	HT treatment at 150 °C for 4 h	FM, C, vacuum drying	pH 8 Borate buffer	330/420	1650 (5500–4.4 × 10^6^)	[34]
C-dots	Pear juice	HT treatment at 150 °C for 2 h	C	Water	360/455	1570 (0–50,000)	[36]
I-C-dots	L-histidine	Electrochemical treatment at pH 9	FM, 500 Da DM for 24 h	pH 3 PBS	420/505	220 (300–3000)	[37]
N-C-dots	Citric acid and L-histidine	Pyrolysis at 220 °C for 2 h in N atmosphere	1000 Da DM	pH 4 NaAc-Hac buffer	360/450	190 (600–30,000)	[32]
N-S-C-dots	[C4mim] [Cys]	Sulfuric acid carbonization at 120 °C for 36 h	N, 500 Da DM	pH 7 PBS	336/430	180 (500–5000)	[14]
PEI-C-dots	Biomass tar and PEI	HT treatment at 180 °C for 2 h	FM, 3500 Da DM for 24 h	pH 4 PBS	340/460	80 (80–400,000)	[6]
C-dots	Leeks	Pyrolysis at 350 °C for 3 h		pH 7.4	360/450	50 (10–104)	[15]
C-dots	Petroleum coke	Ultrasound-assisted chemical oxidation	N, FM, 3500 Da DM for 72 h	EDTA	420/513	29 (250–10,000)	[38]
PA-C-dots	*Vitis vinifera* juice	Thermolysis at 200 °C for 6 h	FM	pH 7 BR	435/498	20 (70–60,000)	[39]
N-C-dots	Pak choi juice	HT treatment at 150 °C for 12 h	FM, C	pH 7.4 Tris-HAc	380/460	10 (0–100)	[10]
TPEA-C-dots	Graphite rods	Electrochemical treatment, TPEA Fun.	FM, C	pH 7 H_2_O/C_2_H_5_OH (9:1, *v*/*v*)	420/500	10 (1000–100,000)	[40]
C-dots	PEI	Microwave-assisted method	N, FM, 1000 Da DM for 24 h	pH 7 BR	360/462	7 (10–2000)	[12]
BPEI-C-dots	Citric acid and BPEI	Pyrolysis for 3 h	SGCC	pH 4	365/460	6 (10–1100)	[16]
C-dots	Pine cones	Microwave pyrolysis at 1000 W for 1 h	C, FM, vacuum drying	pH 4 PBS	360/430	5 (Not linear)	[41]
C-dots	Prawn shells	HT treatment at 200 °C for 8 h	C, vacuum drying	pH 4 PBS	330/405	5 (0.1–5000)	[11]
N-C-dots	Urea and EDTA	Pyrolysis at 200 °C for 1 h	FM	Water	360/434	2.3 (1–22,000)	[42]
PDA-PEI copolymer dots	DA-HCl and BPEIDopamine hydro- Chloride	Polymerization	FM, 1000 Da DM for 4 h	pH 5 PBS	380/530	1.6 (1.6–80,000)	[43]
C-dots	Grass	HT treatment at 180 °C for 3 h	C	pH 7 PBS	360/443	1 (0–50,000)	[44]
S-C-dots	CEW and L-cysteine	Room-temperature base catalysis for 6 h	None	Water	530/559	52 (0–15,700)	This work

λex/λem: excitation/emission wavelengths; BPEI: branched PEI; BR: Britton–Robinson buffer; C: centrifugation; CPDs: C-dots from urine of diet heavily supplemented with vitamin C; Cyclam: Sodium N-cyclohexylsulfamate; Da: Dalton; DA-HCl: Dopamine hydro-Chloride; DM: dialysis membrane; DTPA: 3–3′-dithiodipropanoic acid; EDTA: Ethylenediaminetetraacetic acid; FM: filter membrane; Fun.: Functionalization; LOD: limit of detection; LR: linear range; N: neutralization; NR: not reported; PA-C-dots: polyamine-functionalized C-dots; PDA: polydopamine; PEI: Polyethyleneimine; HT: hydrothermal; I-C-dots: Iodide-C-dots; SGCC: silica gel column chromatography; TPEA: ([N-(2-aminoethyl)-N,N,N0-tris(pyridin-2-ylmethyl) ethane-1,2-diamine]; TTDDA: 4,7,10-trioxa-1,13-tridecane diamine; [C4mim] [Cys]: 1-butyl-3-methylimidazo-lium 2-amino-3-mercaptopropionic acid(L-cysteine).

## Data Availability

The original contributions presented in this study are included in the article. Further inquiries can be directed to the corresponding author.
